# Effect of silicon spraying on rice photosynthesis and antioxidant defense system on cadmium accumulation

**DOI:** 10.1038/s41598-024-66204-9

**Published:** 2024-07-03

**Authors:** Hongxing Chen, Xiaoyun Huang, Hui Chen, Song Zhang, Chengwu Fan, Tianling Fu, Tengbing He, Zhenran Gao

**Affiliations:** 1 College of Agriculture, Guizhou University, Guiyang, 550025 China; 2 Institute of New Rural Development, Guizhou University, Guiyang, 550025 China; 3 Soil Fertilizer Research Institute, Guizhou Academy of Agricultural Sciences, Guiyang, 550025 China; 4 College of Resource and Environmental Engineering, Guizhou University, Guiyang, 550025 China

**Keywords:** Rice, Cd, Si, Photosynthesis, Antioxidant defense system, Plant sciences, Environmental sciences

## Abstract

Cadmium (Cd) pollution is a serious threat to food safety and human health. Minimizing Cd uptake and enhancing Cd tolerance in plants are vital to improve crop yield and reduce hazardous effects to humans. In this study, we designed three Cd concentration stress treatments (Cd1: 0.20 mg·kg^−1^, Cd2: 0.60 mg·kg^−1^, and Cd3: 1.60 mg·kg^−1^) and two foliar silicon (Si) treatments (CK: no spraying of any material, and Si: foliar Si spraying) to conduct pot experiments on soil Cd stress. The results showed that spraying Si on the leaves reduced the Cd content in brown rice by 4.79–42.14%. Si application increased net photosynthetic rate (Pn) by 1.77–4.08%, stomatal conductance (Gs) by 5.27–23.43%, transpiration rate (Tr) by 2.99–20.50% and intercellular carbon dioxide (CO_2_) concentration (Ci) by 6.55–8.84%. Foliar spraying of Si significantly increased the activities of superoxide dismutase (SOD) and peroxidase (POD) in rice leaves by 9.84–14.09% and 4.69–53.09%, respectively, and reduced the content of malondialdehyde (MDA) by 7.83–48.72%. In summary, foliar Si spraying protects the photosynthesis and antioxidant system of rice canopy leaves, and is an effective method to reduce the Cd content in brown rice.

## Introduction

Cadmium (Cd) is a major pollutant affecting the quality of farmland soil, and its biological toxicity is significant^1^. Because of its high solubility and fluidity, the toxic effects of Cd on plants are manifested in various metabolic activities. A large amount of Cd accumulates in plants, leading to a reduction in plant photosynthetic rate, inhibition of plant antioxidant enzyme activity, and suppression of cell division, thereby impacting plant growth^2–4^. Moreover, it enters the human body through the food chain and causes harm^5^. Rice (*Oryza sativa* L.) is the main food crop in China and also the main source of food for over 50% of the global population. However, because of soil pollution, the accumulation of Cd in rice has led to “Cd rice”^6^, and long-term consumption of food contaminated by Cd often induces cancer, pain, kidney toxicity and hypertension^7^. Therefore, the monitoring and control of Cd pollution need to be strengthened to ensure human health and food security.

Many measures have been taken to reduce the accumulation of Cd in rice, including soil remediation works, agricultural practices, and phytoremediation^8^. These remediation techniques are complex and often expensive on a site scale^9^. As the most important foreign nutrient organ of rice, leaves can absorb foreign substances and transport nutrients to other organs of rice^10^. Compared with other agronomic control measures, foliar spraying barrier agent has the characteristics of consistent farming time, convenient application, economical and efficient, and has been widely used in farmland production. It has a good effect on improving crop stress resistance, enhancing crop heavy metal tolerance and increasing crop yield^11^.

Silicon (Si) is not only a beneficial element^12^, the application of Si can promote the absorption of nutrients by plants, significantly enhance their biological and abiotic resistance, which is conducive to plant growth, but also reduce the toxic effect of Cd on plants^13^. Research has found that under Cd stress, Si application increased chlorophyll content (SPAD), carotenoid content and photosynthetic rate of maize leaves^14^. The application of exogenous Si reduced the malondialdehyde (MDA) content of cotton, increased the activity of antioxidant enzymes, alleviated the adverse effects of Cd stress on the growth and photosynthetic characteristics of cotton, and improved the quality of cotton^2^. Foliar Si application significantly increased rice yield, reduced the bioavailability of Cd in soil, inhibited the migration and transformation of Cd in soil and plants, slow down the content of Cd in rice, and improved the quality of rice^15^.

The above researches mainly focus on acid soil or hydroponic experiments, but there are few researches on neutral paddy soil in southern China. Therefore, a pot experiment was conducted to study the effects of foliar Si spraying on growth, photosynthetic characteristics and antioxidant system of Cd-stressed rice in southern rice soil. We proposed the following hypothesis: that foliar Si spraying treatment suppresses the migration and transportation of Cd by rice plants, thereby reducing the Cd content in brown rice, increasing rice yield, improving rice light utilization efficiency, and enhancing antioxidant effects, ultimately enhancing the alleviating effect on Cd toxicity. Therefore, this study aimed to: (1) explore the effect and mechanism of foliar Si application on the migration and accumulation of Cd in rice plants; (2) explore the potential and mechanism of foliar Si application on improving rice photosynthesis and stress resistance; and (3) explore the effect and mechanism of foliar Si application on enhancing the antioxidant capacity of rice. The results will provide a valuable reference for reducing the accumulation of Cd in rice, improving its safety as food, and ensuring human health.

## Materials and methods

### Experimental design

The experiment was conducted at the Guiyang Comprehensive Experimental Station of the Guizhou Academy of Agricultural Sciences in China from March to October 2021 (106°39′20″E, 26°29′59″N). The pot experiment was conducted under natural sunlight and temperature (from March to October, 2022). The air temperature ranged between 10.5 ± 5.6 and 28.6 ± 2.5 °C. The relative humidity varied from 77 ± 2.8 to 93 ± 0.5%. The basic physical and chemical indicators of soils are shown in Table 1.

**Table 1:** Basic physical and chemical indicators of soils.

Property	Measuring method	Value
pH	Potentiometric method	6.58
Organic matter (g·kg^−1^)	Potassium dichromate external heating method	115.01
Hydrolyzable nitrogen N (mg·kg^−1^)	Sulfuric acid digestion sodium salicylate method	49.25
Available P (mg·kg^−1^)	Sulfuric acid digestion molybdenum antimony colorimetric method	5.96
Available K (mg·kg^−1^)	Ammonium acetate extraction flame photometric method	153.90

The background value of soil Cd was 0.20 mg·kg^−1^, and 0.20 mg·kg^−1^ was the risk screening value for Cd in the rice fields. The experiment set up three exogenous Cd concentration addition treatments: Cd1 (0), Cd2 (0.20 mg∙kg^−1^), and Cd3 (0.40 mg∙kg^−1^). The rice variety used was “Jingliangyou 534” (Guoshen Rice 20,176,004). Each box was uniformly inserted in four holes as a treatment, with two plants per hole, and each treatment was replicated six times for a total of 18 pots. Before transplanting the rice seedlings, base fertilizer was applied: 450 kg·hm^−2^ rice-specific compound fertilizer. Tillering fertilizer (urea 120 kg·hm^−2^) was sprayed onto the plants during the tillering stage, and potassium chloride fertilizer (112.5 kg·hm^−2^) was sprayed onto the plants during the booting stage. The soil type was yellow loamy paddy soil. Adding of exogenous Cd to the soil involved mixing Cd chloride (CdCl_2_) (Shanghai Aladdin biochemical technology Co., Ltd., Shanghai, China) in solution with air-dried soil, injecting water to saturation, and equilibrating for 4–5 weeks. The actual values after addition were Cd1 (0.20 mg·kg^−1^), Cd2 (0.60 mg·kg^−1^), and Cd3 (1.60 mg·kg^−1^). When the rice was mature, the SPAD, Cd content, photosynthetic parameters, and enzyme activity of rice canopy leaves under different Cd concentrations were measured. Two foliar spraying treatments: CK (no spraying of any material) and Si (foliar spraying of Si) were used. In the spraying of “Jianggeling” rice with foliar Si fertilizer (Foshan Ironman Environmental Technology Co., Ltd., Foshan, China), the active ingredients were primarily high-purity SiO_2_ sols (Si ≥ 85 g·L^−1^, pH = 5.0–7.0) at a concentration of 2.5 g·L^−1^. One spray was administered at the jointing stage and one at the heading stage, at 17:00–18:00 p.m.

### Measurement of indicators

#### Determination of Cd content

An inductively coupled plasma optical emission spectrometer (ICP-OES, Thermo Fisher Scientific, Waltham, MA, USA) was used to measure the Cd content^16^. At the maturity stage of rice, we handpicked three robust and evenly developed specimens from every group. Subsequently, these specimens underwent multiple cleansings using faucet water, followed by a deionized water purge. Post-washing, the samples were subjected to a brief heat exposure at 105 °C for half an hour and subsequently desiccated in a heating chamber regulated at 75 °C to achieve a stable mass. Afterward, the samples were methodically segmented into the roots, stems, leaves, husks, and brown rice parts, and were finely pulverized with the assistance of a high-velocity FW-100 grinder (Tianjin Taist Instrument Co., Ltd.). Subsequently, a 200 mg portion of the rice specimen was measured out, and to this, we introduced 5 mL of nitric acid (HNO_3_). The digestion process for the specimen was carried out with a graphite digestion device at a temperature of 120 °C for a duration of two hours, proceeding until no residual sediment remained within the digestion chamber. The temperature was adjusted to 150 °C to evaporate the acid. The sample was removed and allowed to cool, diluted to a volume of 50 mL in a volumetric flask, filtered, and analyzed via ICP–OES.

#### Measurement of photosynthetic parameters

Portable photosynthetic apparatus (GFS-3000, Heinz Walz GmbH, Bavaria, Germany) was used to measure the photosynthetic parameters of rice at the heading stage^17^. Between 10:00–11:00 a.m. on a clear, cloudless day, the carbon dioxide (CO_2_) concentration was set to 400 µ mol·mol^−1^, the light intensity was set to 1200 µ mol·m^−2^·s^−1^, the air velocity was set to 0.5 L·min ^−1^, leaf temperature was 25 °C, and relative humidity was set to 70%. We handpicked three robust and evenly developed specimens from every group to measure the net photosynthetic rate (Pn), transpiration rate (Tr), stomatal conductance (Gs), and intercellular CO_2_ concentration (Ci).

#### Measurement of fluorescence parameters

A portable fluorometer (Junior-PAM, Heinz Walz GmbH, Bavaria, Germany) was used to measure chlorophyll fluorescence parameters^18^. We selected rice leaves with consistent growth conditions, subjected to fully adapt to darkness for 30 min, and measured the maximum photochemical quantum yield of photosystem II (PS II) of the leaves, maximum photochemical efficiency (Fv/Fm), actual photochemical efficiency (Y(II)), initial fluorescence (Fo), and non-photochemical quenching coefficient (NPQ).

#### Measurement of MDA and antioxidant enzymes

We cleaned rice leaves with distilled water and weighed 100 mg of fresh rice leaves, ground them into a homogenate using liquid nitrogen in a mortar and pestle, and then transferred the homogenate to a 4 mL centrifuge tube. We added 1 mL of 0.05 mol·L^−1^ phosphate buffer (pH 7.8) to the tube and fixed the volume to 4 mL. This was mixed well using a vortexer, and was put into a frozen high-speed centrifuge at 4 °C and 10,000 r·min^−1^ for 10 min, The supernatant was then placed in a refrigerator at 4 °C as a backup. The activities of superoxide dismutase (SOD) and peroxidase (POD) in rice leaves and the MDA content were determined using the nitroblue tetrazolium (NBT) photoreduction method^19^, guaiacol method, and thiobarbituric acid (TBA) method, respectively. All measurements were performed using enzyme activity assay kits from Wuhan PureBiochemical Co., Ltd.

#### Determination of relative chlorophyll content

We used a portable chlorophyll meter (SPAD-502 Plus, Minolta, Tokyo, Japan) to measure the SPAD value of leaves in situ^20^. When they had been measured, we selected three rice plants with uniform growth conditions and, for each plant, selected an intact leaf and measured the SPAD value at the central position six times, and took the average value as the SPAD value for that point. When taking measurements, we avoided areas with concentrated veins and used appropriate shading to block direct sunlight, to ensure the accuracy of the measurement.

### Data processing and analysis

The bioconcentration factor (BCF) of Cd in rice was calculated (1) as^21^:

1 $${\text{The BCF of Cd in various parts}} = \frac{{\text{Cd content in various parts}}}{{\text{Cd content in soil}}}$$

The transport factor (TF) of Cd in rice was calculated (2) as^22^:

2 $${\text{TF}}_{{{\text{A}} - {\text{B}}}} = \frac{{\text{Cd content in A}}}{{\text{Cd content in B}}}$$

where TF refers to the ratio of heavy metal concentration in part A to that in part B of the rice plant.

Data processing and statistical analysis were carried out using SPSS 24.0 (IBM Corp., Armonk, NY, USA). Single factor analysis of variance (ANOVA) was used to tested the same treatment under different Cd concentrations. The treatment effects at different Cd concentrations were compared by using the least significant different test with the *P* value < 0.05. The effects with Si treatment and CK treatment were tested by t test. Plots were generated using Origin.

## Results

### Effect of foliar spraying Si on Cd content in various organs and on rice yield

The impact of foliar spraying Si on the Cd content in various organs and on rice yield are shown in Table 2.

**Table 2:** Effects of foliar spraying of Si on Cd content of various organs of rice and rice yield.

Cd Treatment	Cd content in various parts of rice(mg^.^kg^–1^)						Yield(kg^.^ha^–1^)
	Root	Stem	Leaf	Cob	Husk	Brown rice	
Cd1							
CK	0.431 ± 0.039c	0.063 ± 0.009a	0.020 ± 0.002c	0.027 ± 0.004b	0.024 ± 0.003ab	0.044 ± 0.010a	4369.4 ± 157.6ab
Si	0.918 ± 0.058b	0.074 ± 0.004a	0.026 ± 0.005b	0.041 ± 0.005a	0.017 ± 0.001b	0.026 ± 0.011b	4552.1 ± 116.9a
Cd2							
CK	1.137 ± 0.153b	0.071 ± 0.002b	0.053 ± 0.003ab	0.044 ± 0.005b	0.048 ± 0.004c	0.109 ± 0.019a	4144.6 ± 76.6a
Si	1.259 ± 0.114b	0.174 ± 0.022a	0.057 ± 0.007a	0.071 ± 0.004a	0.028 ± 0.005b	0.103 ± 0.011ab	4242.5 ± 207.2a
CK	2.639 ± 0.409a	0.601 ± 0.040a	0.102 ± 0.006b	0.164 ± 0.013b	0.118 ± 0.010a	0.167 ± 0.011a	3654.9 ± 234.2ab
Si	2.931 ± 0.081a	0.611 ± 0.026a	0.129 ± 0.004a	0.267 ± 0.018a	0.078 ± 0.008b	0.146 ± 0.008b	3879.3 ± 229.5a

Cd1, Cd2, Cd3: three Cd concentration stress treatments (Cd1: 0.20 mg·kg^-1^, Cd2: 0.60 mg·kg^−1^, and Cd3: 1.60 mg·kg^−1^). CK, Si: two foliar Si treatments (CK: no spraying of any material, Si: foliar Si spraying). Data present the mean ± standard deviation of three replicates. Lower case letters indicate significant differences (*p* < 0.05).

Under the three Cd concentrations, the Cd content in each organ of rice increased with the increase in Cd concentration. At Cd1 concentration, compared with CK, Si treatment increased the Cd content in roots, leaves, and cobs by 112.99%, 30.00%, and 51.85%, respectively, and reduced the Cd content in husks and brown rice by 29.17% and 40.91%, respectively. At Cd2 concentration, compared with CK, Si treatment significantly increased the Cd content in the stems and cobs by 145.07% and 61.36%, respectively, and significantly decreased the Cd content in husks by 41.67%, respectively. At Cd3 concentration, compared with CK, Si treatment increased the Cd content in leaves and cobs by 26.47% and 62.80%, respectively, and decreased the Cd content in husks and brown rice by 33.90% and 12.57%, respectively. Rice yield decreased with increasing Cd concentration. At the three Cd concentrations, spraying Si on the leaves increased rice yield by 4.18%, 2.36%, and 6.14% compared to CK, respectively. It can be seen that Cd stress will increase the Cd content in various organs and reduce rice yield. In contrast, spraying Si on the leaves can change the accumulation of Cd in various organs, and increase rice yield.

### Effect of foliar spraying Si on the accumulation and transport of Cd in rice

**Figure 1:**
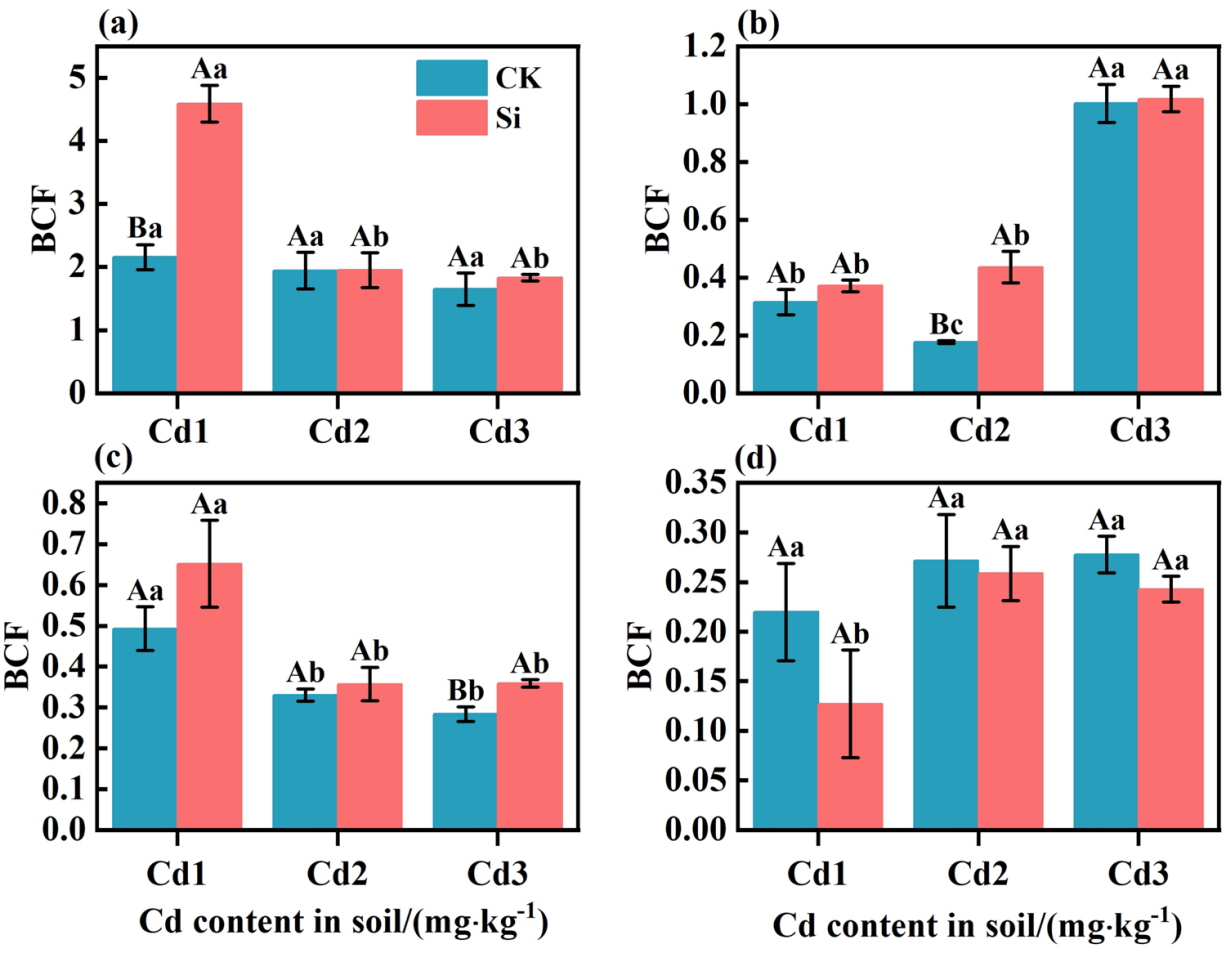
The effect of foliar Si spraying on the BCF of various organs in rice: BCFroot (**a**), BCFstem (**b**), BCFleaf (**c**), BCFbrown rice (**d**). Cd1, Cd2, Cd3: Three Cd concentration stress treatments (Cd1: 0.20 mg·kg^−1^, Cd2: 0.60 mg·kg^−1^, and Cd3: 1.60 mg·kg^−1^). CK, Si: two spraying treatments (CK: no spraying of any material, Si: foliar Si spraying). Data present the mean ± standard deviation of three replicates. Capital letters indicate significant differences (*p* < 0.05) between CK and Si treatments at the same Cd concentration. Lowercase letters indicate significant differences (*p* < 0.05) between CK or Si treatments at different Cd concentrations.

The effect of foliar spraying Si on the BCF in various organs are shown in Fig. 1. The accumulation of Cd in rice showed a trend of root > stem > leaf > brown rice. Under CK treatment, the enrichment coefficients of roots, stems, leaves, and brown rice were 1.65–2.16, 0.18–1.00, 0.28–0.49, and 0.22–0.28, respectively. Compared with CK, under treatments Cd1, Cd2, and Cd3, foliar application of Si enhanced rice BCFroot by 112.91%, 0.41%, and 11.03%; BCFstem by 17.78%, 146.33%, and 1.60%; BCFleaf by 32.25%, 8.18%, and 26.41%; and decreased rice BCFbrown rice by 42.27%, 4.78%, and 12.59%, respectively. It can be seen that foliar application of Si can increase the enrichment coefficients of rice roots and leaves, which decreased the Cd enrichment coefficients of brown rice.

**Figure 2:**
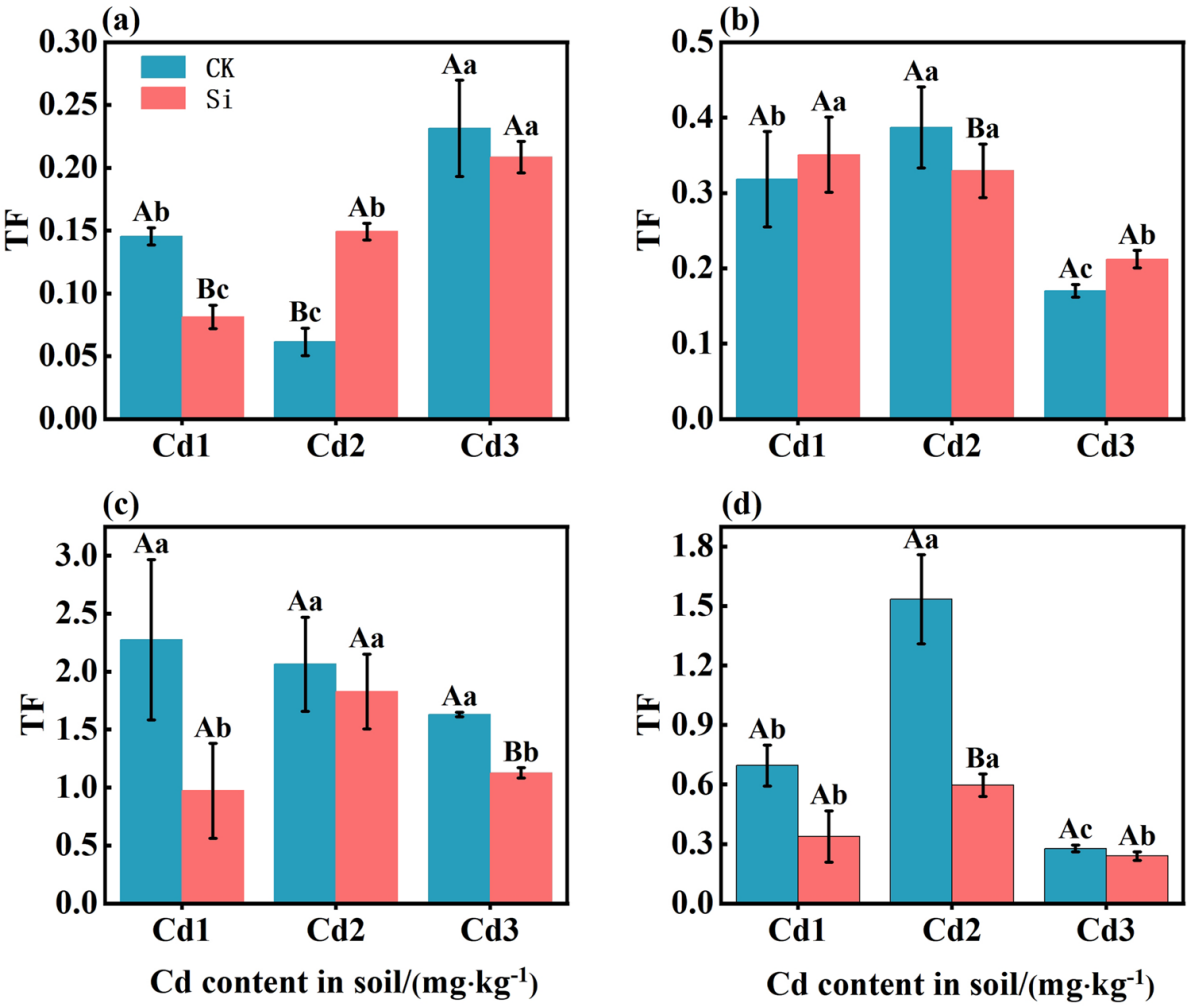
The effect of foliar Si spraying on the TF of various organs in rice: TFroot-stem (**a**), TFstem-leaf (**b**), TFleaf-brown rice (**c**), TFstem-brown rice (**d**). Cd1, Cd2, Cd3: Three Cd concentration stress treatments (Cd1: 0.20 mg·kg^−1^, Cd2: 0.60 mg·kg^−1^, and Cd3: 1.60 mg·kg^−1^). CK, Si: two spraying treatments (CK: no spraying of any material, Si: foliar Si spraying). Data present the mean ± standard deviation of three replicates. Capital letters indicate significant differences (*p* < 0.05) between CK and Si treatments at the same Cd concentration. Lowercase letters indicate significant differences (*p* < 0.05) between CK or Si treatments at different Cd concentrations.

The effect of foliar spraying Si on the TF in various organs are shown in Fig. 2. Foliar spraying of Si decreased TFleaf-brown rice, and TFstem-brown rice. Compared with CK, foliar spraying of Si decreased rice TFleaf-brown rice by 57.27%, 11.43%, and 30.90%, and of TFstem-brown rice by 51.44%, 61.15%, and 15.90%. It can be seen that foliar application of Si can decrease the transfer coefficient of rice leaf to brown rice and stem to brown rice, which decreased the Cd content of brown rice.

### Effect of foliar spraying Si on the SPAD values of rice leaves

**Figure 3:**
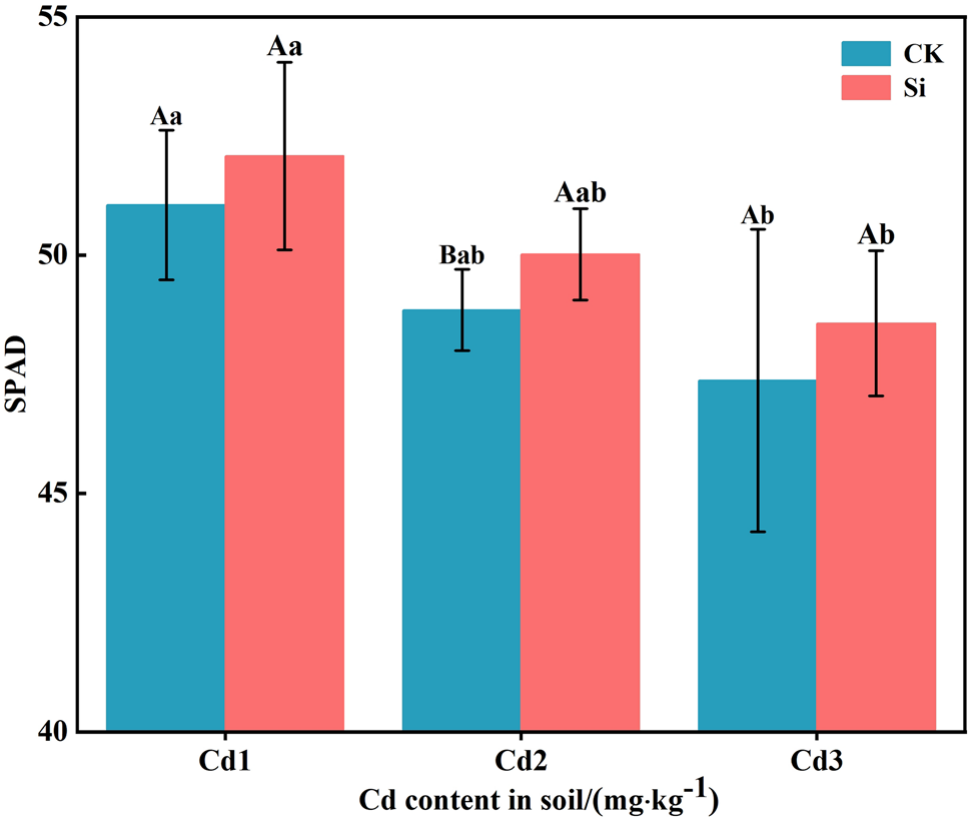
The effects of foliar Si spraying on the SPAD values of rice leaves. Cd1, Cd2, Cd3: Three Cd concentration stress treatments (Cd1: 0.20 mg·kg^−1^, Cd2: 0.60 mg·kg^−1^, and Cd3: 1.60 mg·kg^−1^). CK, Si: two spraying treatments (CK: no spraying of any material, Si: foliar Si spraying). Data present the mean ± standard deviation of six replicates. Capital letters indicate significant differences (*p* < 0.05) between CK and Si treatments at the same Cd concentration. Lowercase letters indicate significant differences (*p* < 0.05) between CK or Si treatments at different Cd concentrations.

The effect of foliar spraying Si on the SPAD values of rice leaves are shown in Fig. 3. The SPAD values of leaves under three Cd concentration treatments all decreased with the increase in Cd concentration. Compared with the control Cd1 treatment, the decrease in Cd2 and Cd3 treatments was 4.31% and 7.22%, respectively. Under the treatment of spraying Si, the decrease in Cd2 and Cd3 treatments was 3.97% and 6.75%, respectively, compared with the treatment Cd1. Foliar spraying of Si resulted in an increase of 2.02–2.53% in SPAD values compared to the control group. Thus it can be seen that Cd reduces the SPAD values of rice leaves, whereas foliar spraying of Si enhances these values.

### Effect of foliar spraying Si on photosynthetic parameters of rice leaves

**Figure 4:**
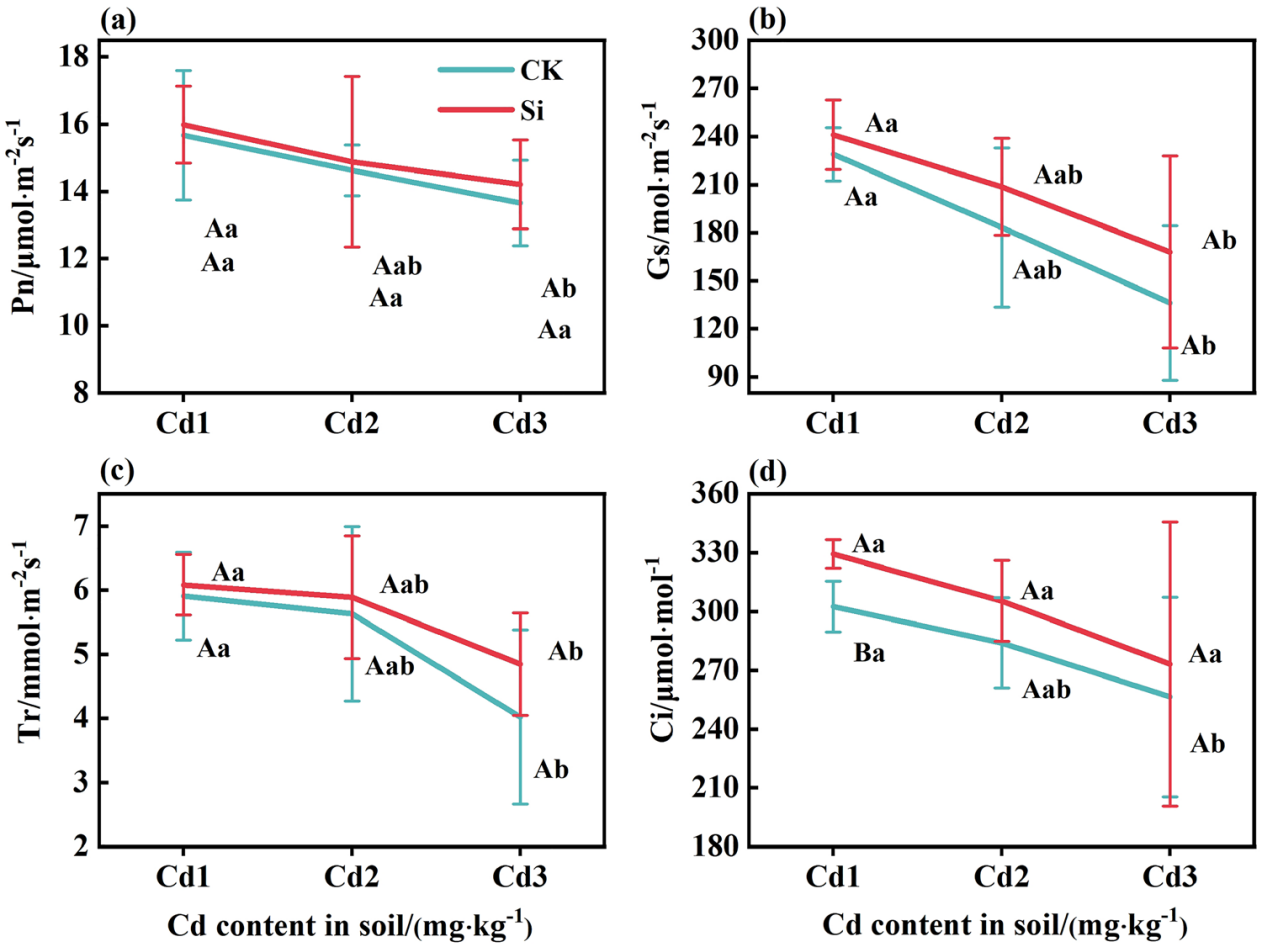
The effects of foliar Si spraying on the photosynthetic parameters of rice leaves: net photosynthetic rate (Pn) (**a**), stomatal conductance (Gs) (**b**), transpiration rate (Tr) (c), intercellular CO_2_ concentration (Ci) (d). Cd1, Cd2, Cd3: Three Cd concentration stress treatments (Cd1: 0.20 mg·kg^−1^, Cd2: 0.60 mg·kg^−1^, and Cd3: 1.60 mg·kg^−1^). CK, Si: two spraying treatments (CK: no spraying of any material, Si: foliar Si spraying). Data present the mean ± standard deviation of three replicates. Capital letters indicate significant differences (*p* < 0.05) between CK and Si treatments at the same Cd concentration. Lowercase letters indicate significant differences (*p* < 0.05) between CK or Si treatments at different Cd concentrations.

The impact of foliar spraying Si on the photosynthetic parameters of rice leaves are shown in Fig. 4a–d. As Cd concentration increased, under CK and Si treatment, the Pn, Gs, Tr, and Ci values all decreased. Under CK treatment, compared with Cd1 concentration, the Pn, Gs, Tr, and Ci at Cd2 concentration decreased by 6.68%, 19.97%, 4.65%, and 6.13%, respectively. In contrast, at the concentration of Cd3, the decrease amplitude was 12.86%, 40.54%, 31.88%, and 15.25%, respectively. Compared to CK, foliar spraying Si resulted in a 1.77–4.08% increase in Pn, a 5.27–23.43% increase in Gs, a 2.99–20.50% increase in Tr, and a 6.55–8.84% increase in Ci, respectively. Therefore, Cd diminished the photosynthetic attributes of rice, whereas foliar spraying Si can mitigate the toxic impact of Cd on rice, enhance its the photosynthetic parameters, and foster photosynthesis.

### Effect of foliar spraying Si on the fluorescence parameters of rice leaves

**Figure 5:**
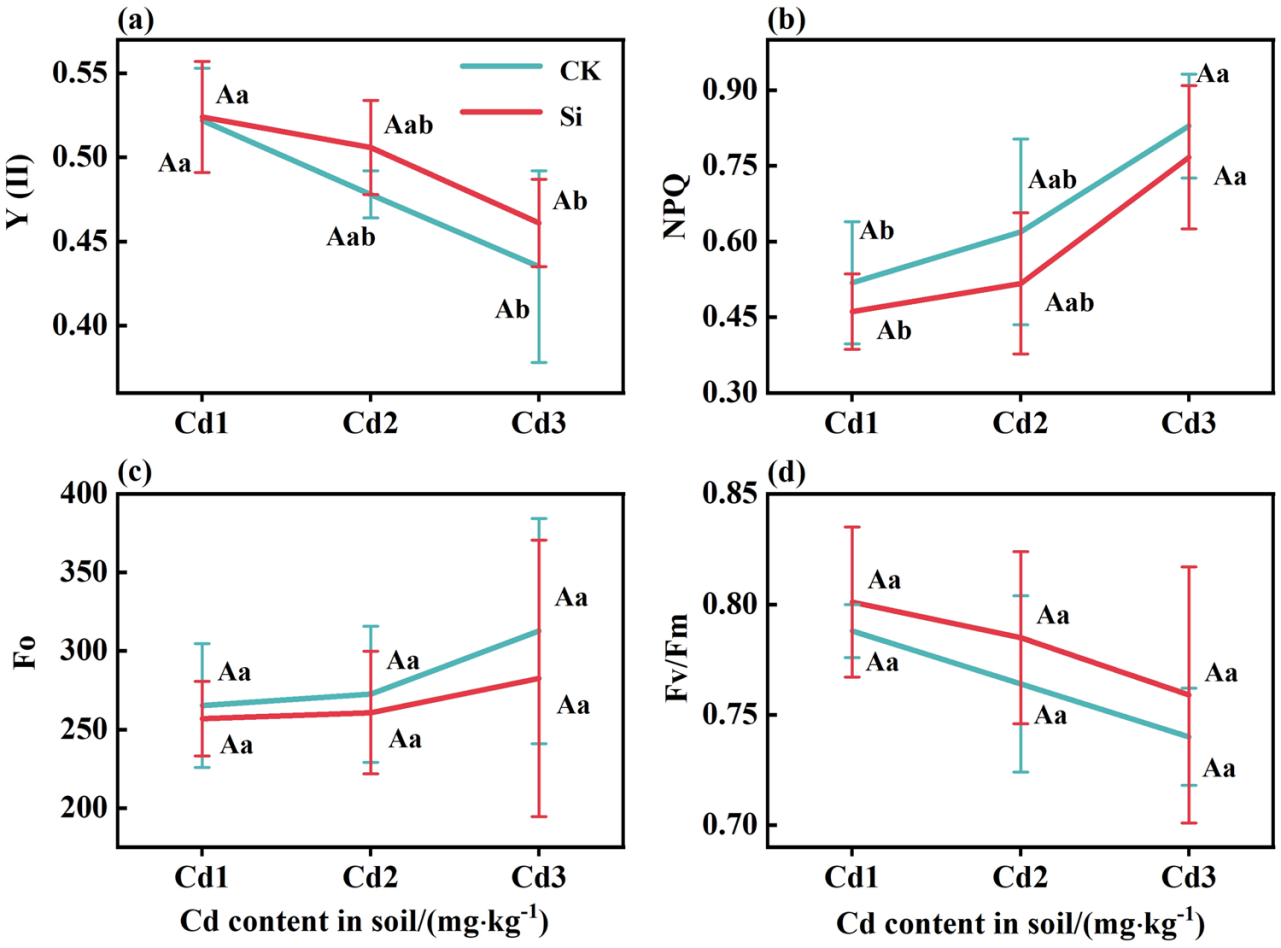
The effects of foliar Si spraying on the chlorophyll fluorescence parameters of rice leaves: actual photochemical efficiency: Y (II) (**a**), non photochemical quenching coefficient: (NPQ) (**b**), initial fluorescence (Fo) (**c**), maximum photochemical efficiency (Fv/Fm) (**d**). Cd1, Cd2, Cd3: Three Cd concentration stress treatments (Cd1: 0.20 mg·kg^−1^, Cd2: 0.60 mg·kg^−1^, and Cd3: 1.60 mg·kg^−1^). CK, Si: two spraying treatments (CK: no spraying of any material, Si: foliar Si spraying). Data present the mean ± standard deviation of three replicates. Capital letters indicate significant differences (*p* < 0.05) between CK and Si treatments at the same Cd concentration. Lowercase letters indicate significant differences (*p* < 0.05) between CK or Si treatments at different Cd concentrations.

The influence of foliar spraying Si on the fluorescence parameters of rice leaves are shown in Fig. 5a–d. where Y(II) and Fv/Fm decreased with increasing Cd concentration, whereas Fo and NPQ increased with increasing Cd concentration. At the three concentrations of Cd1, Cd2, and Cd3, compared to CK, foliar Si spraying resulted in an increase in Y(II) and Fv/Fm by 0.38–5.98% and 1.55–2.78%, respectively, while simultaneously reducing Fo and NPQ by 3.11–9.67% and 7.48–16.47%, respectively. Under Cd stress, foliar application of Si can effectively maintain high photosynthetic characteristics.

### Effect of foliar spraying Si on MDA, POD, and SOD content in rice leaves

**Figure 6:**
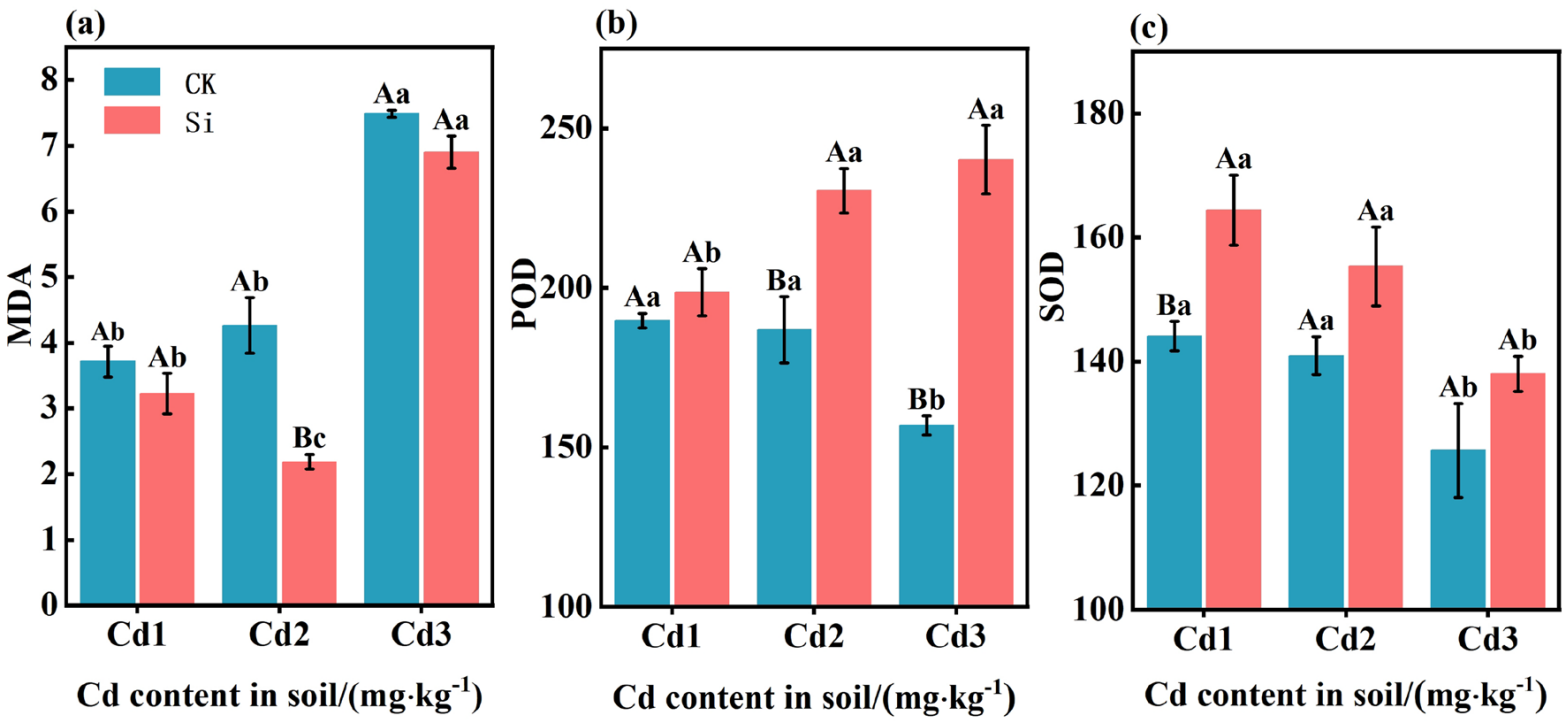
The effects of foliar Si spraying on MDA, POD and SOD content in rice leaves: malondialdehyde: MDA (**a**), peroxidase: (POD) (**b**), superoxide dismutase (SOD) (**c**). Cd1, Cd2, Cd3: Three Cd concentration stress treatments (Cd1: 0.20 mg·kg^−1^, Cd2: 0.60 mg·kg^−1^, and Cd3: 1.60 mg·kg^−1^). CK, Si: two spraying treatments (CK: no spraying of any material, Si: foliar Si spraying). Data present the mean ± standard deviation of three replicates. Capital letters indicate significant differences (*p* < 0.05) between CK and Si treatments at the same Cd concentration. Lowercase letters indicate significant differences (*p* < 0.05) between CK or Si treatments at different Cd concentrations.

The impact of foliar spraying Si on the levels of MDA, POD, and SOD activity are shown in Fig. 6a–c. Under CK treatment, as the concentration of Cd increased, the content of MDA rose, whereas the activities of POD and SOD decreased. Compared to CK, foliar spraying of Si significantly reduced the content of MDA in rice leaves by 7.83–48.72%. Additionally, compared to CK, foliar spraying of Si significantly increased the activities of SOD and POD in rice leaves by 9.84–14.09% and 4.69–53.09%, respectively. It can be seen that foliar Si application can improve the antioxidant enzyme activity of rice leaves.

## Discussion

The excessive accumulation of Cd in plants not only significantly impacts their growth, development, quality, and yield, but also poses a potential threat to human health through the food chain because of its concealed presence within the plants^23^. The half-life of Cd in the human body is up to 30 years and it has a cumulative effect^24^. Excess Cd accumulation in the human body will cause calcification of kidney and bone, metabolic dysfunction, bone pain, hypertension, diabetes, emphysema, and other diseases. It can lead to deoxyribonucleic acid (DNA) oxidative damage and inhibition of the repair path, and induce the generation of cancer cells^25^. Research has shown that Si can effectively reduce the harm of Cd to plants and its inhibitory effect on plant growth, thereby improving the beneficial effects of food safety and human health^26^. In this study, foliar Si application had no significant effect on rice yield, but there was a trend of increasing yield under different soil Cd concentrations (Table 2). This is consistent with previous research findings^27^. The application of Si under metal stress can improve plant growth by increasing nutrient elements, SPAD, root volume, organic acid secretion, and histological characteristics^28–30^. Cd was highest in the roots of rice plants (Table 2), consistent with previous research findings^31,32^. The root system is the first plant organ to sense adversity. Excessive Cd can cause a decrease in the number of roots, a shortening or browning of roots, a decrease in root area, and can affect the division of root tip cells, inducing stress Chromosome aberrations and other factors can significantly reduce the absorption capacity of roots for water and nutrients in the soil^33^. Overall, the high retention of Cd in roots is considered a defense mechanism for plants to alleviate metal stress^27^. The Cd content in brown rice is a major health risk of significant concern^33^. The results showed that Si application could significantly reduce the Cd content in brown rice (Table 2) (*P* < 0.05). Under Cd stress in brown rice, Si application significantly reduced the transfer and enrichment coefficients of Cd, and reduced the Cd content^34^. The application of Si on the leaves mainly reduces the accumulation of Cd in brown rice by inhibiting the migration of Cd from the stem to the rice grains (Fig. 1). The stem is the main organ that restricts the transport of Cd to rice^35^. The stem nodes of Poaceae plants are the hub for the distribution of mineral elements to different organs, and the upward transport of Cd is significantly restricted at these nodes^36^. The high Cd content in the cell wall of stems and leaves is due to the presence of a large number of negatively charged functional groups in the cell wall^37^. These functional groups are precipitated and complexed with positively charged heavy metal ions, allowing most of the Cd to bind to the cell wall. Rice can alleviate the toxic effects of Cd by combining Cd and Si in the cell wall to alter the redox potential^38^. Combining with Si in the form of negatively charged hemicellulose can inhibit the absorption of Cd by rice cells. Therefore, foliar spraying of Si fertilizer is a feasible method to control Cd accumulation in rice grains, thereby reducing its risk to human health through the food chain.

In higher plants, Cd affects photosynthesis mainly by reducing the SPAD values, causing a decrease in the content of photosynthetic pigments, disrupting the position of matrix layers and grana within chloroplasts, leading to a decrease in the photosynthetic capacity of chloroplasts^39^. Cd can also inhibit the enzymes related to photosynthesis and affect plant growth by altering transpiration, respiration, and stomatal switch, thereby inhibiting crop photosynthesis^40^. This study found that, under Cd stress, the SPAD in rice leaves decreased (Fig. 3), this is consistent with the previous studies^39^. On the one hand, this is because Cd accumulates in rice leaves, altering the ultrastructure of chloroplasts, severely damaging the thylakoid membrane and chloroplasts, and leading to a decrease in SPAD^41^. On the other hand, because the peroxidation reaction produces a large amount of hydrogen peroxide (H_2_O_2_), this enters the chloroplast through the plasma membrane, attacks the chloroplast pigment protein complex, and inhibits the activity of plant chlorophyll ester reductase, and SPAD reduction is caused by factors such as chlorophyll degradation^39,42^. The gas exchange parameters (Ci and Tr) are limiting factors for CO_2_ diffusion and immobilization, and are related to the activities of CO_2_ immobilized enzymes, ribulose diphosphate carboxylase, and oxygenase (RuBisCO)^43^. The toxicity of Cd can be mediated by increasing the carboxylation efficiency of RuBioCO^44^. In this study, the photosynthetic parameters Pn, Gs, Tr, and Ci of rice decreased with increasing Cd concentration (Fig. 4). This is consistent with previous reports on Cd inhibiting plant photosynthesis^45^, where low Cd concentrations significantly inhibited plant growth and photosynthesis in rice and mustard^45,46^. The inhibition of photosynthesis induced by Cd is usually attributed to the inhibition of key enzyme activities in the Calvin cycle and photosynthetic electron transport chain^47^, and this negative effect can be alleviated through the supply of Si. When Si is sprayed on the leaves, the plant toxicity of Cd is reduced, and the inhibition of Cd on photosynthesis is reduced, thereby improving the performance of photosynthesis. Pn is a determining factor in plant growth^48^. In this study, the increase in Pn value after foliar Si spraying treatment may be attributed to the increase in Gs and Tr, which accelerates the effective carbon assimilation period of rice leaves and thus accelerates the accumulation of photosynthetic products. Therefore, Si can increase the SPAD value of rice leaves and improve photosynthesis^39^.

The changes in chlorophyll fluorescence can reflect biotic or abiotic stress^49^. The decrease in Fv/Fm and Y (II) indicates that the toxicity of Cd inhibits the photoactivation of PSII, which is due to the destruction of antennal pigments, limited electron transfer from PSII to photosystem I (PSI), and disruption of the integrity of the thylakoid membrane structure. The decrease in Fo (Fig. 5c) means that the potential efficiency of PSII has undergone a negative change, and an decrease in NPQ (Fig. 5b) indicates an improvement in the efficiency of photochemical reactions. In this experiment, foliar application of Si resulted in more light energy absorbed by rice plants being used for photochemical reactions and energy or carbohydrate synthesis, thereby increasing quantum yield and protecting the photosynthetic system from damage. These findings are also consistent with the results of rice photosynthetic parameters.

Cd does not participate in redox reactions in cells, but can induce the formation of reactive oxygen species (ROS) in plants^50^. Although the increase in ROS synthesis in cells poses a threat to cellular biomolecules, ROS also acts as a signaling molecule, activating stress response and defense-related genes through signaling pathways^51^. In this study, the increase in ROS production level under Cd stress was manifested as an increase in MDA content (Fig. 6a), a decrease in SPAD (Fig. 3), and a decrease in leaf photosynthetic gas exchange (Fig. 4). These findings are consistent with previous studies^52,53^. In previous research findings, Cd toxicity was found to have a negative impact on various physiological, biochemical, and metabolic processes in plants^2,54^. Research has shown that the toxicity of Cd to maize can induce the production of H_2_O_2_ and MDA^55^. However, the mediation by Si can reduce the final product of lipid peroxidation, namely the MDA content, which helps to reduce membrane permeability and maintain its integrity^56^. Under Cd toxicity, various enzymatic and non-enzymatic antioxidant defense systems are activated to control the production of ROS. Enzyme antioxidants, including SOD , POD, and catalase (CAT), are another defense system. SOD converts superoxide radicals into H_2_O_2_, which appears in plant tissues as a result of Cd stress. H_2_O_2_ is a powerful oxidant that accumulates in plant tissues through SOD channelization reactions. It is blocked by the circulation of ascorbic acid glutathione. In addition to H_2_O_2_, another toxic oxide is heme oxygenase-1 (OH-1), which can react with all large molecules. SOD can prevent the formation of OH-1 in plant tissues^57^. POD can alter ROS levels in plants due to its role in consuming and clearing H_2_O_2_. Unlike SOD, POD has a high affinity for H_2_O_2_. However, POD can convert H_2_O_2_ into H_2_O and oxygen (O_2_)^58^. In this study, Si application significantly increased the activity of POD and SOD (Fig. 6b and c) (*P* < 0.05). This is consistent with previous research results, which showed that Si application increased SOD activity in wheat and sorghum plants^50,54^. Si application increases POD activity in wheat leaves under Cd stress^59^. Similarly , Si treatment reduces the production of ROS and promotes enzymatic and non-enzymatic antioxidants for ROS clearance^60^.

Cd exposure can cause a range of harmful effects on organisms, including humans. Therefore, understanding the mechanisms of Cd uptake, translocation and accumulation in rice is important for strengthening strategies to effectively reduce Cd. In the future, the effect of foliar Si spraying on Cd accumulation at stem nodes and internodes of rice must be further enhanced. It can both efficiently control the transfer of Cd to the critical part of the grain and reduce the Cd contamination of rice in the soil.

## Conclusion

This study demonstrated that Cd stress increased the Cd content in rice roots, stems, and leaves, decreased the SPAD of rice leaves and photosynthetic efficiency of rice leaves, inhibited the activities of SOD and POD in rice leaves, increased the MDA content in rice leaves, and inhibited rice growth. After applying Si, the Cd content of brown rice can be reduced, the SPAD of rice leaves can be increased, the photosynthetic characteristics of rice leaves can be improved, the SOD and POD activities of rice leaves can be increased, the MDA content of rice leaves can be reduced, and rice yield can be promoted.

### Experimental research and feld studies on plants statement

In the study only cultivated plants were used which are neither endangered nor at risk of extinction. We confrm that their handling was performed in compliance with relevant institution, national and international guidelines and legislation.

### Supplementary Information

Supplementary Information.

## Data Availability

Data is provided within the manuscript or supplementary information files.
